# Effect of Anatomic Contextual Information on the Performance of a Convolutional Neural Network Tasked With Brain Metastasis Detection

**DOI:** 10.1016/j.adro.2026.102092

**Published:** 2026-05-25

**Authors:** Ethan Truong, Jordan Houri, Gretchen Hermann, Vitali Moiseenko, Jona Hattangadi-Gluth, Jeffrey Rudie, Aaron B. Simon

**Affiliations:** a Department of Radiation Medicine and Applied Sciences, University of California San Diego, La Jolla, California; b Indiana University School of Medicine, Indianapolis, Indiana; c Weldon School of Biomedical Engineering, Purdue University, West Lafayette, Indiana; d Department of Radiology, Scripps Health, San Diego, California; e Department of Radiation Oncology, University of California Irvine, Irvine, California

## Abstract

**Purpose:**

Modern management of brain metastases requires close surveillance with serial magnetic resonance imaging, creating interest in techniques for automated detection. The convolutional neural network (CNN) is currently the dominant approach to automated metastasis detection, and multiple variations of the CNN have been investigated to improve performance. In this work, we looked beyond the impact of network architecture and assessed the impact on performance of providing anatomic contextual information to a CNN during the training period.

**Methods and Materials:**

The nnU-Net, a widely adopted CNN, was selected for this study. The nnU-Net was trained on an institutional data set comprising 301 high-resolution, T1-weighted, contrast-enhanced magnetic resonance images and associated structure labels consisting of both target brain metastases (1111 total) and several organs at risk (OARs) that were labeled in the process of radiosurgery planning. During the training period, the model was presented with either labeled brain metastases (BM) alone or with the complete structure set of metastases and OARs (BM+OAR). The test set contained 100 cases and 421 total metastases.

**Results:**

We found that, by multiple standard performance metrics, the BM+OAR model outperformed the BM model. Median case-wise Dice coefficient for BM was 0.75 (IQR, 0.51-0.86) and for BM+OAR was 0.79 (IQR, 0.58-0.86) (*P* < .001). Median sensitivity for BM was 1.0 (IQR, 0.71-1.0) and for BM+OAR was 1.0 (IQR, 0.75-1.0) (*P* < .01). Median positive predictive value for BM was 0.5 (IQR, 0.25-0.74) and for BM+OAR was 0.77 (IQR, 0.50-1.0) (*P* < .001). Importantly, among lesions scored as “false-positives,” 47/151 detected by BM+OAR could be retrospectively verified to be real tumors, whereas only 22/231 detected by BM could be verified to be real (*P* < .001).

**Conclusions:**

These findings suggest that providing anatomic contextual information can improve the accuracy of CNN-based automated brain metastasis detection algorithms and open new opportunities for research into the optimization of automated detection approaches for brain metastases.

## Introduction

Brain metastases are a common manifestation of metastatic cancer, affecting approximately one-third of patients. Historically, brain metastases conferred a poor prognosis and limited lifespan.^1^ Treatment options were limited in the absence of effective systemic therapies and local therapies, such as surgery and whole brain radiation, prolonged life only in select circumstances.^2^^,^^3^

Over the last 2 decades, improvements in systemic and radiation therapy techniques have significantly improved the prognosis and treatment-related morbidity of patients with brain metastases.^4^, ^5^, ^6^ However, intracranial relapses are common^5^, ^6^, ^7^ and must be closely monitored. Consequently, regular imaging surveillance of patients with brain metastases has become the standard of care.

Imaging surveillance is a time- and labor-intensive process entailing meticulous slice-by-slice review of high-resolution contrast-enhanced magnetic resonance imaging (MRI) volumes. New lesions must be identified from a background of stable lesions, treatment-related effects, and vascular structures. False-positive identification of new lesions can lead to unnecessary treatments or changes in systemic therapy. Conversely, false-negative identification may delay necessary care.

To improve the accuracy and efficiency of imaging surveillance, multiple groups have designed machine learning algorithms to detect brain metastases from MRIs.^8^, ^9^, ^10^, ^11^, ^12^, ^13^, ^14^, ^15^, ^16^, ^17^, ^18^, ^19^ Among the most successful approaches have been convolutional neural networks (CNN), which are readily applicable to image-based identification problems. Efforts to organize the testing of these algorithms against large, standardized data sets are ongoing and demonstrate promise for improving CNN performance.^20^

Beyond the development of better algorithms and standardized training data sets, there may be other opportunities to improve the performance of brain metastasis detection algorithms, opportunities that cannot be explored with standardized data sets. In this work, we asked whether providing anatomic contextual information to a CNN-based metastasis detection model during training would impact its performance. Specifically, we asked whether simultaneously training the algorithm to detect metastases and several normal cranial structures would affect its performance when compared with training for metastasis detection alone. The availability of a single-institutional data set derived from linear accelerator-based radiosurgery treatment plans, in which several normal tissue structures are routinely contoured for treatment planning purposes, provided a window of opportunity to perform this proof-of-concept analysis.

## Methods and Materials

### Data set and image preprocessing

As part of an institutional review board-approved study, 401 radiosurgery treatment plans from 265 distinct patients treated between 2011 and 2018 at a single institution were identified retrospectively from the institution’s treatment planning platform. From each treatment plan, the three-dimensional (3-D), thin-cut, T1-weighted gadolinium contrast-enhanced brain MRI volume used for treatment planning was extracted in DICOM (Digital Imaging and Communications in Medicine) format and anonymized. Gross tumor volumes for lesions undergoing treatment and planning target volumes (PTVs) for previously treated metastases were extracted. In addition, an institutional standard set of organs at risk (OARs) was extracted. The standard set of OARs included the brainstem, optic nerves and chiasm, the globes, and the lenses of the eye. Because these structures had been contoured for clinical purposes, all were contoured by practicing radiation oncologists with specialization in stereotactic radiosurgery.

Because the MRI volumes included in this study were initially used for clinical purposes over the course of several years, the data set includes images derived from multiple 1.5T and 3T scanners from various manufacturers. Although the exact postgadolinium 3D volumetric T1-weighted inversion recovery spoiled gradient-recalled sequence image acquisition parameters varied, the typical acquisition matrix size was 512 × 512 × 216, with a voxel size of 0.5 × 0.5 × 1.0 mm.

Image volumes were randomly separated into training and test data sets, with distinct patients in the training and test sets. The training data set consisted of 301 brain stereotactic radiosurgery cases and 1111 metastases. The test set contained 100 cases and a total of 421 metastases. There were 81 distinct patients in the test data set. The median and modal number of cases per patient was 1. The maximum number of cases per patient was 6. For each case, new brain metastases were labeled distinctly from previously treated brain metastases, which were indicated by prior PTVs as described above.

### Convolutional neural network

The nnU-Net^21^ was selected as the CNN to employ for this study. The nnU-Net was designed to standardize the process by which decisions pertaining to image preprocessing, network architecture modification, hyperparameter tuning, training, and postprocessing are made. Although the nnU-Net does not uniformly outperform other deep learning-based image segmentation algorithms, it has been shown to be competitive with cutting-edge deep learning pipelines across a wide range of medical image segmentation challenges. Within the realm of brain metastasis detection, nnU-Net has been shown by several groups to perform well by several standard metrics of segmentation quality.^8^^,^^22^ The nnU-Net was selected to minimize the potential impact of model selection decisions on our findings and isolate, as much as possible, the impact of providing anatomic contextual information. The code for implementing the nnU-Net is freely available to the public at https://github.com/MIC-DKFZ/nnUNet. The code used to run the nnU-Net for the purposes of this study, as well as the model weights, have been uploaded to an online repository (https://data.mendeley.com/preview/jhxrwsct49?a=1ded0970-a36f-42c6-99b6-ca4bca6a91aa).

DICOM MRI volumes and binary masks derived from target and OAR structures (background, metastasis, brainstem, globes, lenses, optic chiasm, and optic nerves) were converted to 3D Neuroimaging Informatics Technology Initiative files. Resulting training and testing data sets (imaging and label volumes) were input into the nnU-Net planning and preprocessing routine as described by the developers.^21^ The details of the nnU-Net processing pipeline are described in detail elsewhere.^21^ However, briefly, the nnU-Net is designed to be automatically self-configuring with the intention of eliminating the need for either manual optimization of or ad hoc implementation of numerous design and parameter selection choices that can significantly impact CNN performance. The nnU-Net thus automatically makes decisions about image preprocessing parameters (eg, voxel size and spacing as well as intensity distribution), model architecture (eg, two-dimensional vs 3D UNet), and training parameters (eg, batch and patch sizes, loss function, data augmentation, and number of training epochs). The output of the nnU-Net is a volume of predicted voxel-wise labels for each image volume in the test data set. Because the goal of this project was not to optimize or assess the performance of the nnU-Net itself, we intentionally did not attempt to provide any manual input into the nnU-Net parameter selection process.

The nnU-Net was trained using 2 approaches. With both approaches, the model was presented with the same image volumes in the training and testing data sets. For the first approach, the model was presented with only the segmented brain metastases as label volumes. This model was termed BM. For the second approach, the model was presented with metastasis label volumes and OAR label volumes (BM+OAR). Both nnU-Net v1 3d_fullres models used the same automatically determined data set-specific preprocessing and network planning, despite differing label sets. For both models, nnU-Net selected a target voxel spacing of 1.0 × 0.5 × 0.5 mm, applied non-computed tomography intensity normalization to both input channels without foreground masking, and used a 3D patch size of 64 × 192 × 160 voxels with batch size 2. Thus, the only difference between the 2 models was the number of output labels, whereas preprocessing and core training configuration remained unchanged. Models were run on an NVIDIA RTX 3090 graphics card.

### Model performance evaluation

To assess the performance of the model in predicting brain metastases, several metrics were calculated. The first metric was the Dice similarity coefficient (DSC). DSC measures the similarity or overlap between the ground truth (GT) and predicted segmentations (PS). It is defined as

$$\text{DSC}=\;\frac{2\;|GT\cap PS|}{|GT|+|PS|}$$

where |GT∩PS| is the number of voxels designated as belonging to a brain metastasis in both the GT and PS, and |GT| an |PS| are numbers of voxels separately belonging to brain metastases in the GT and PS volumes, respectively. The DSC can be performed either case-wise, in which a single score is calculated for the entire volume, or lesion-wise, in which a DSC is calculated separately for each lesion and then averaged over a case to provide an average DSC for each case. The lesion-wise approach was adopted for the 2023 Brain Tumor Segmentation (BraTS) Segmentation Challenge,^20^ given its reduced bias toward models that capture only large lesions. The lesion-wise approach can be described by the formula

$$\text{DS}C_{lesion-wise}=\;\frac{\sum_{i}^{L}DSC\left(l_{i}\right)}{TP+FN+FP}$$

where $DSC(l_{i})$ is the DSC for each GT lesion, *L* is the number of GT lesions, and TP, FP, and FN are the detected number of true-positive, false-positive, and false-negative lesions, respectively. The FP term in the denominator is intended to penalize false-positive lesions. Freely available code for segmentation of individual lesions and calculation of lesion-wise metrics was developed for the 2023 BraTS challenge and was used for this calculation (https://github.com/rachitsaluja/BraTS-2023-Metrics). Here, both the case-wise and lesion-wise scores are reported.

In addition, the 95% percentile of the Hausdorff distance (HD95), a metric describing the maximum difference in boundary contour between 2 shapes, was calculated both case-wise and lesion-wise using the approach developed for the 2023 BraTS challenge.^20^ Using this approach, the lesion-wise HD95 is calculated for each TP lesion. For each FP and FN lesion, an HD95 of 374 mm is assigned. The final calculation is then:

$$\text{HD}95_{lesion-wise}=\;\frac{\sum_{i}^{TP}HD95\left(l_{i}\right)+374*FN+374*FP}{TP+FN+FP}$$

Note that in absolute terms, the lesion-wise HD95 metric will yield much higher numbers than the case-wise metric, as it highly penalizes both false-positive and false-negative lesions, even if they are small.

Sensitivity was calculated as the number of true-positive metastases as a fraction of the total GT metastases for each case. The positive predictive value was calculated as the fraction of detected metastases that were true positives for each case.

The concordance correlation coefficient (CCC) between predicted and GT lesion volume was calculated both lesion-wise and case-wise. For the lesion-wise analysis, only the GT lesions were included (eg, a false-positive predicted lesion is not included). For the case-wise analysis, the full GT and predicted tumor volumes are included, which include the effects of false-positive predicted lesions.

Statistical comparisons across groups were performed using the paired Wilcoxon signed-rank test for continuous metrics and the chi-squared test for frequency metrics. The visualization of image segmentations was generated with ITK-SNAP.^23^

## Results

### Size and distribution

In total, 1111 metastases from 301 MRI scans were segmented in the institutional training data set, and 421 metastases from 100 MRI scans were segmented in the test data set. The average number of brain metastases per patient was similar between the training set (3.7 ± 4.2) and the test set (4.2 ± 4.7) (*P* = .74). The average volume of contrast-enhancing lesions was similar between the training (1.54 ± 7.2 cm^3^) and test data set (1.48 ± 4.24 cm^3^; *P* = .85).

### Detection and segmentation performance

**Figure 1:**
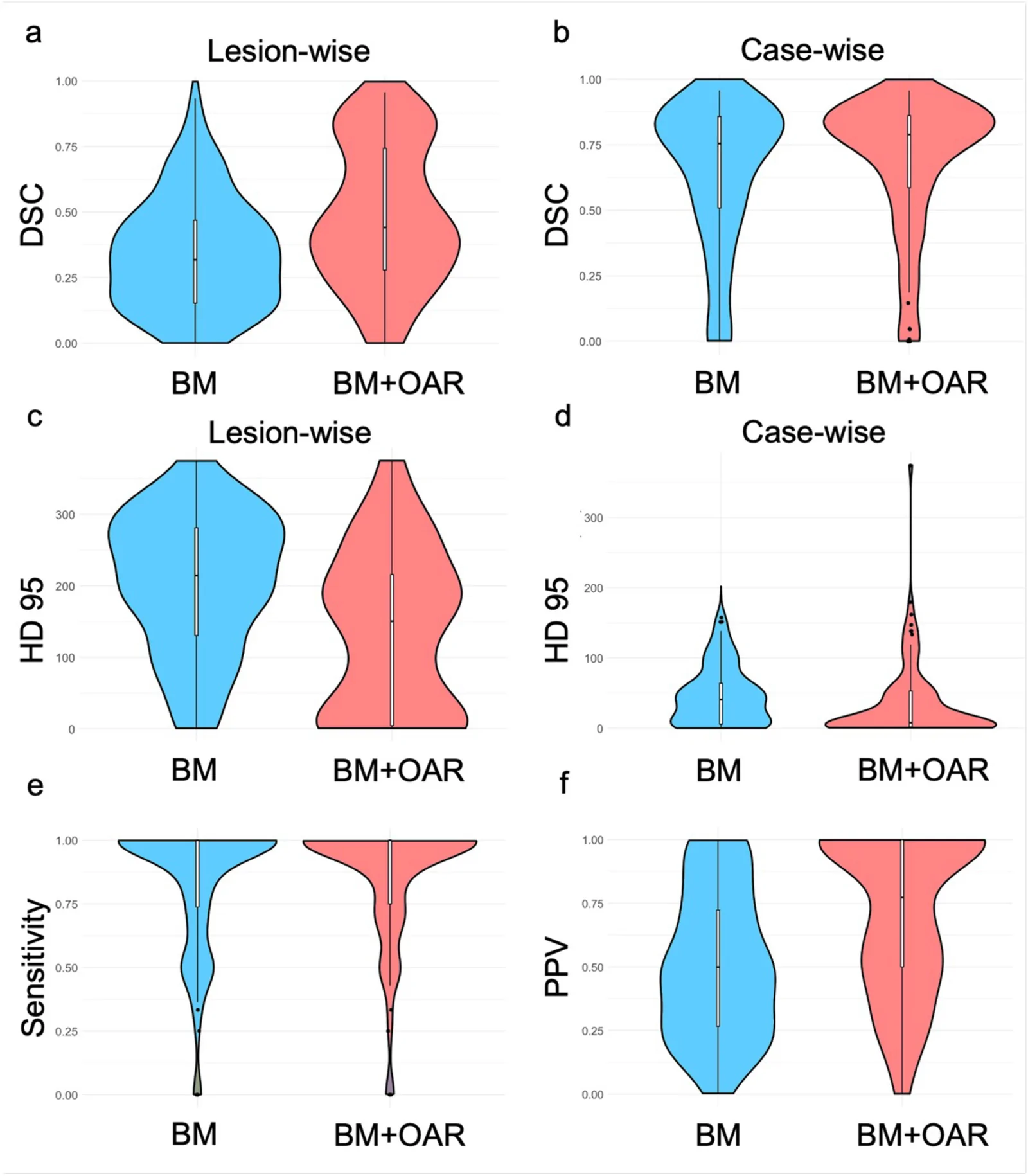
Violin plots demonstrate the distribution of segmentation performance metrics. (a) Lesion-wise Dice similarity coefficient (DSC), (b) case-wise DSC, (c) lesion-wise HD95, (d) case-wise HD95, (e) sensitivity, and (f) positive-predictive value (PPV). *Abbreviations*: BM = brain metastases; HD95 = 95% percentile of the Hausdorff distance; OAR = organ at risk.

**Figure 2:**
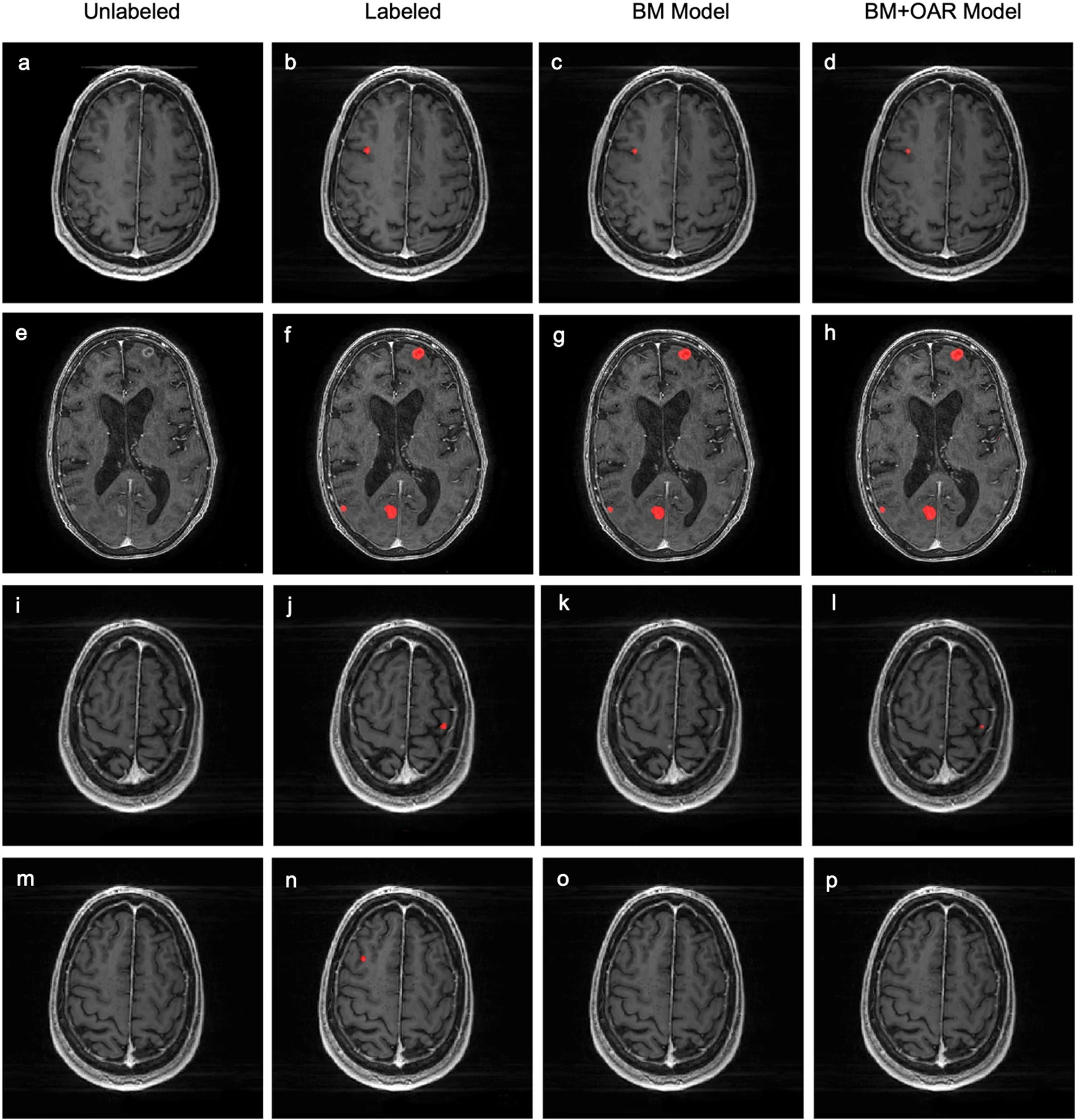
Representative examples of true-positive and false-negative detections. (a-d) A small lesion identified by both the BM and BM+OAR models, (e-h) lesions of varying sizes identified by both the BM and BM+OAR models, (i-l) a lesion correctly detected by the BM+OAR model but missed by the BM model, (m-p) a faint lesion missed by both the BM+OAR and BM models. *Abbreviations*: BM = brain metastases; OAR = organ at risk.

**Figure 3:**
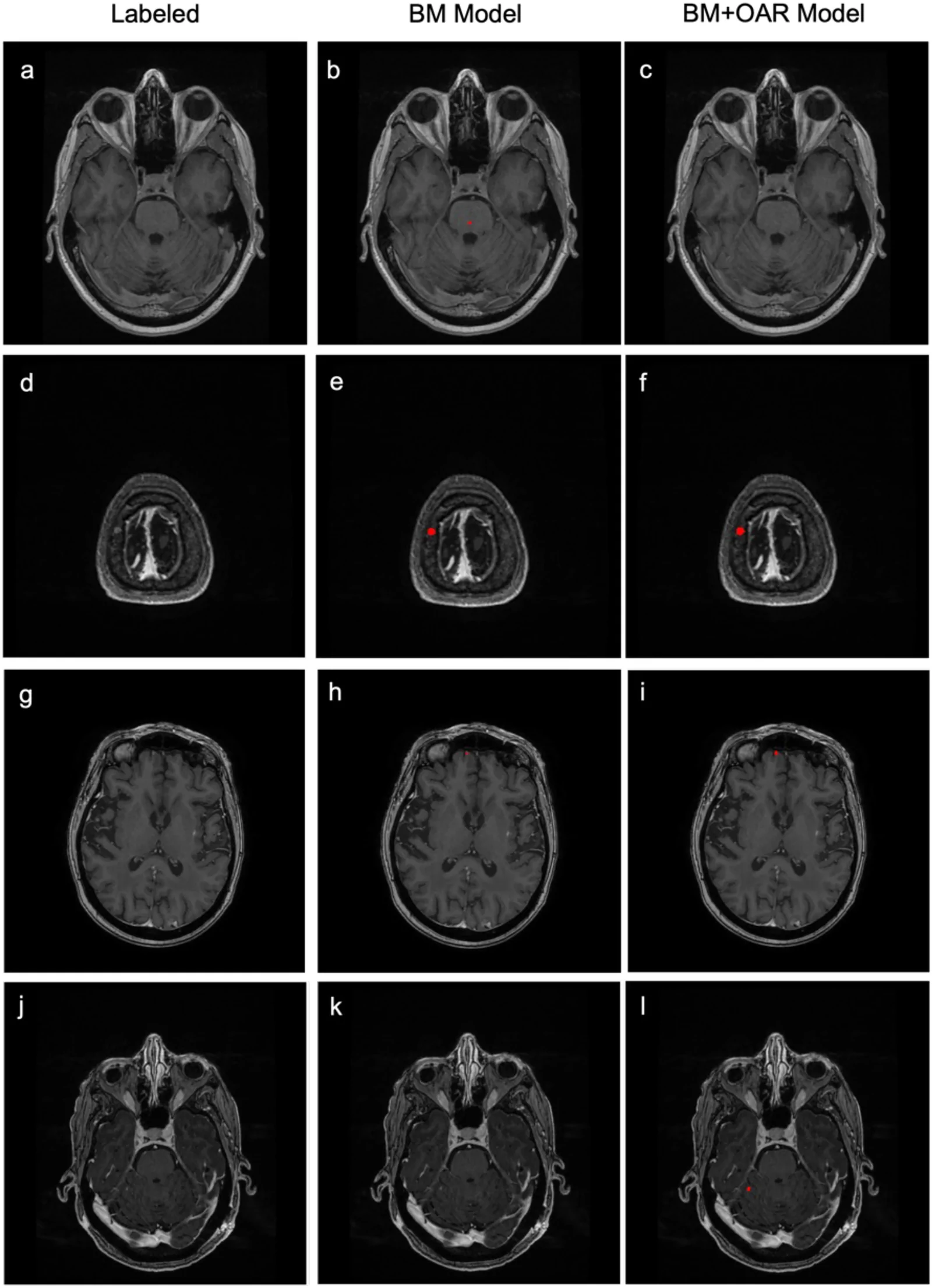
Representative false-positive identifications. (a-c) A region in the brainstem spuriously labeled as a lesion by the BM model, (d-f) a lesion in the skull mislabeled as a brain metastasis by both BM and BM+OAR models, (g-i) an unlabeled lesion identified by the BM and BM+OAR models which grew on a subsequent MRI, (a-c) an unlabeled lesion identified by the BM+OAR but not the BM model which grew on a subsequent MRI. *Abbreviations*: BM = brain metastases; MRI = magnetic resonance imaging; OAR = organ at risk.

The memory footprint of the segmentation pipeline was approximately 8 GB. The average inference time was approximately 20 seconds. The BM+OAR model outperformed the BM model across all calculated metrics, both case-wise and lesion-wise (Table 1 and Fig. 1). Figure 2 demonstrates representative examples of true-positive and false-negative detections, and Fig. 3 demonstrates representative examples of false-positive detections from each of the models.

**Table 1:** Performance of the BM and BM+OAR models

	Performance median (IQR)		
Performance metric	BM	BM+OAR	*P* value
DSC—lesion	0.32 (0.15-0.47)	0.44 (0.27-0.75)	<.001
DSC—case	0.75 (0.51-0.86)	0.79 (0.58-0.86)	<.001
HD95—lesion (mm)	214 (189-374)	150 (4-219)	<.001
HD95—case (mm)	40 (5.8-63.9)	7.7 (2.0-53.0)	<.001
Sensitivity	1.0 (0.71-1.0)	1.0 (0.75-1.0)	<.01
Positive predictive value	0.5 (0.25-0.74)	0.77 (0.50-1.0)	<.001

*Abbreviations*: BM = Brain Metastases; DSC = Dice similarity coefficient; HD95 = 95% percentile of the Hausdorff distance; OAR = organ at risk.

Discrepancies between the lesion-wise and case-wise HD95 and DSC metrics suggested that model errors consisted predominantly of false-positive and false-negative detections of relatively small lesions, which are heavily penalized by the lesion-wise HD95 and DSC but not by the corresponding case-wise metrics.

**Figure 4:**
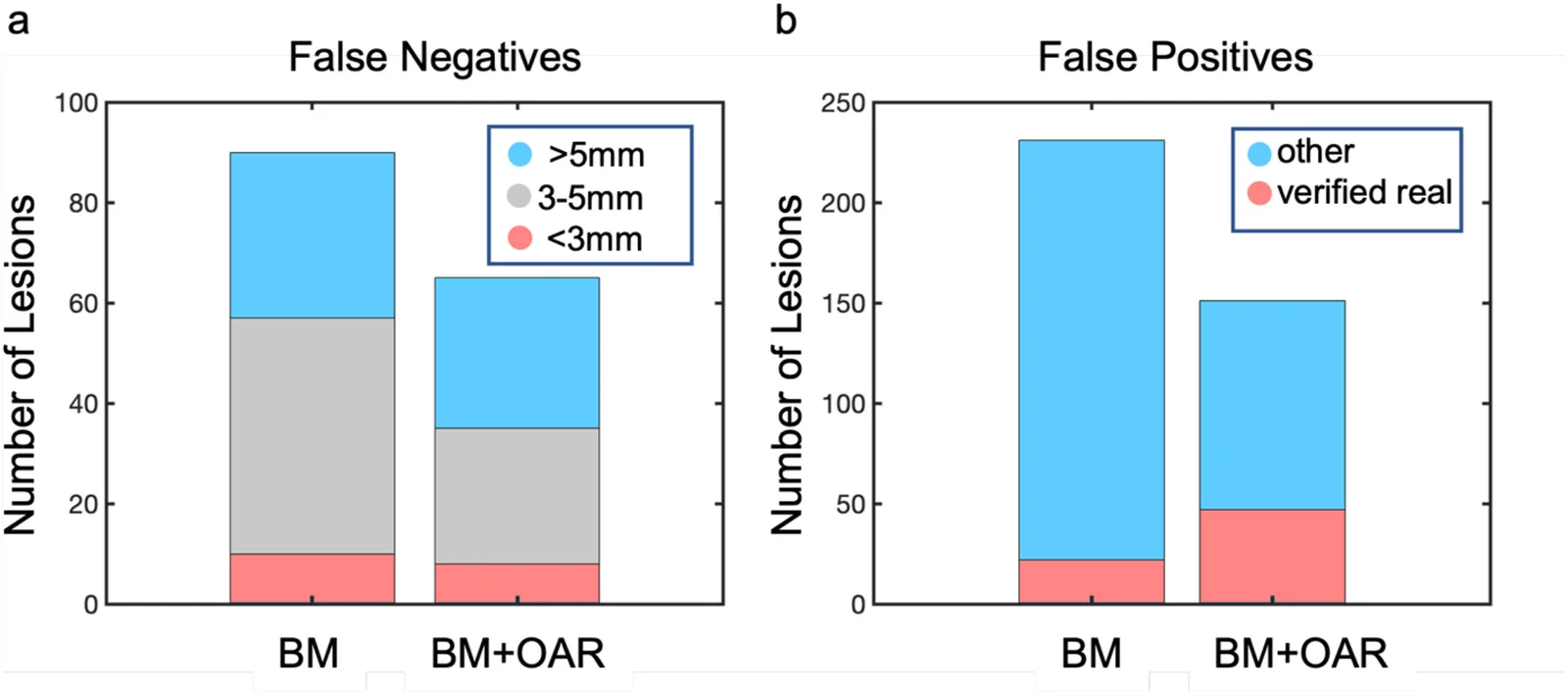
Quantitative analysis of false-positive and false-negative lesions. (a) The BM model missed more lesions than the BM+OAR model, though the size distribution of false negatives was similar. (b) The BM model identified more false-positive lesions than the BM+OAR model, with a higher percentage of those identified by the BM+OAR model later verified to be real lesions. *Abbreviations*: BM = brain metastases; OAR = organ at risk.

This finding prompted a more detailed analysis of false-negative and false-positive detections. There were more false-negative detections for the BM model than the BM+OAR model (Fig. 4a), consistent with the significantly higher sensitivity of the BM+OAR model. The size distribution of false-negative lesions was not significantly different between the 2 models, with similar proportions of missed lesions >5 mm, 3-5 mm, and <3 mm in diameter (*P* = .4).

There were also more false-positive detections for the BM model than the BM+OAR model (Fig. 4b), consistent with the significantly higher positive predictive value of the BM+OAR model. Close analysis of false-positive lesions led to the finding that some lesions designated as false positives were real tumors that were not contoured in the process of radiosurgery planning. In some cases, these were tumors previously treated by whole brain radiation and therefore not associated with a PTV delineation. In some cases, these were very small lesions that were not delineated at the time of radiosurgery planning. For the BM model, 22 (9.5%) false-positive lesions could retrospectively be definitively designated as real tumors in the sense that they could be linked either to prior scans and history of whole brain radiation or to future scans showing interval growth. For the BM+OAR model, 47 (31%) false-positive lesions could be definitively designated as real tumors (*P* < .001, chi-squared test). Other common false positives included lesions that appeared real but could not be definitively verified due to absence of past or future scans (21 for BM+OAR vs 2 for BM), lesions within the calvarium (18 for BM+OAR vs 16 for BM), foci outside the brain without corresponding lesions (21 for BM+OAR vs 49 for BM), intracranial vessels (13 for BM+OAR vs 17 for BM), and other false positives in the brain parenchyma for which no corresponding structure could be identified (31 for BM+OAR vs 125 for BM).

**Figure 5:**
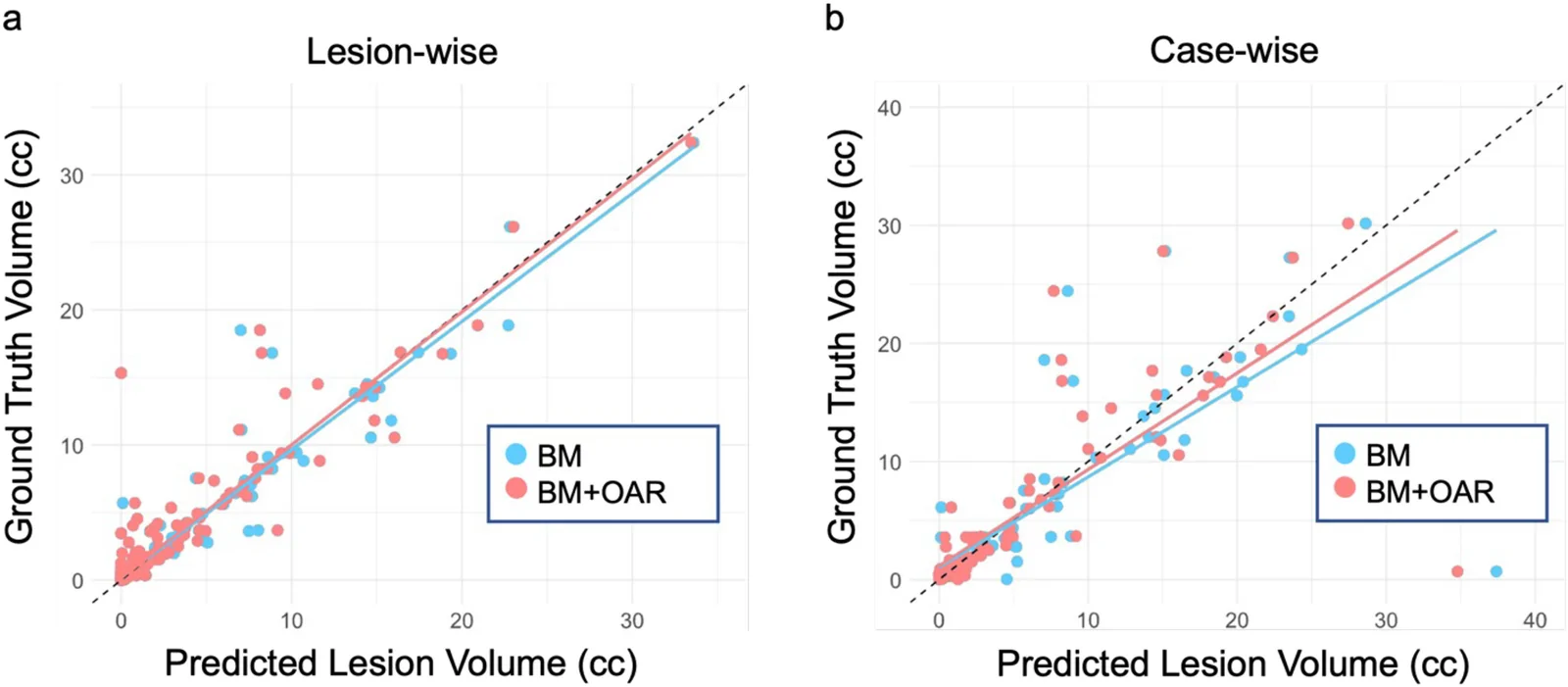
The concordance correlation coefficient between true and predicted lesion volume. (a) Lesion-wise and (b) case-wise. *Abbreviations*: BM = brain metastases; OAR = organ at risk.

The CCC between true and predicted lesion volumes for the BM+OAR and BM models was highly similar. For the lesion-wise analysis, CCC was 0.95 (95% CI, 0.94-0.96) for BM+OAR and 0.95 (95% CI, 0.94-0.96) for BM (Fig. 5a). For the case-wise analysis, CCC was 0.87 (95% CI, 0.81-0.91) for BM+OAR and 0.85 (95% CI, 0.80-0.90) for BM (Fig. 5b).

Assessing performance in segmenting the OARs was not the focus of this work; however, the DSC, HD95, sensitivity, and specificity for each OAR are reported in Table E1. Representative labeled and predicted OAR segmentations from the BM+OAR model are depicted in Fig. E1.

## Discussion

Over the last decade, significant strides have been made in the development of algorithms capable of detecting brain metastases automatically from clinical MRI data sets, which has the potential to significantly change the workflow for physicians who care for patients with brain metastases and to improve patient outcomes. The development of the convolutional neural network greatly advanced this field, and organized efforts to test new algorithms against standardized data sets offer a promising approach to optimizing network architecture. Still, other important questions cannot be answered with standardized data sets, and here we took advantage of a uniquely labeled data set to assess, in a proof-of-principle fashion, the question of whether the presentation of anatomic contextual information assists in the detection of brain metastases. To accomplish this, we trained a well-described CNN, the nnU-Net, on a data set of high-resolution contrast-enhanced MRIs labeled with both brain metastases and a standard set of OARs. Across multiple standard performance metrics, we found that training the model to detect both metastases and OARs improved performance over training the model to detect metastases alone. Although it is inherently challenging to compare the performance of these models with that of other segmentation pipelines because of differences in data sets, segmentation tasks, and performance metrics, the performance of the BM+OAR model was within the range of the scores achieved by entrants to the 2023 BraTS-METS challenge for lesion-wise Dice, lesion-wise HD95, sensitivity, and positive-predictive value.^20^

Importantly, not only did the BM+OAR model perform better than the BM model according to standard metrics, but among lesions deemed formally to be false positives, more lesions found by the BM+OAR model were subsequently determined to be real tumors that were simply not contoured in the course of clinical care. This suggests that this approach may be particularly helpful in the detection of very small lesions, which is a persistent challenge in the field.

This is not the first effort to improve segmentation performance through the introduction of contextual information, although, to our knowledge, it is the first to do so in this clinical situation. Tampu et al^24^ assessed whether providing anatomic masks segmenting gray matter, white matter, and cerebral spinal fluid to an nnU-Net model improved performance in glioma segmentation. Overall, they did not find significant performance gains; however, they did note some improvement when they restricted the imaging modalities available for training and testing to contrast-enhanced T1-weighted MRIs. Kao et al^25^ enriched a standard MRI-based training data set with automatically segmented masks from the Harvard-Oxford brain parcellation atlas and found that adding brain parcellation information improved segmentation performance for gliomas. Others have investigated the addition of symmetry information^26^ and spectral brain coordinate information^27^ into segmentation algorithms to improve performance. In this work, the set of structures chosen to provide contextual information was selected primarily because it was readily available from an existing annotated data set. This work should thus be looked at as a proof of the principle that contextual information can aid in brain metastasis segmentation rather than as a fully optimized approach. Indeed, significant further work will be required to determine whether a subset of these structures is primarily responsible for the performance gains found here and whether additional performance gains may be achieved through the delineation of additional normal structures.

Strengths of this work include pragmatic use of a clinical data set derived from a heterogeneous collection of MR scanners and imaging protocols, potentially increasing the external validity of the result. An additional strength was the employment of a well-accepted modeling pipeline, minimizing the probability that the result was influenced by architectural design, preprocessing steps, or parameter selection choices that would hinder replication of the result. Finally, the in-depth analysis of false-positive lesions addressed a potential drawback of using a clinically annotated data set that can itself contain some annotation errors. Other groups have conducted similar analyses, with similar findings.^8^ A limitation of this work is the use of a relatively small, single-institutional data set. This limited our ability to perform robust subset analyses to investigate whether a smaller set of the OARs we used would have provided equivalent or even superior results. The use of a single institutional data set may also limit the external validity of the approach to data sets from other institutions in which contouring or imaging practices may differ. Validation of this approach against other institutional data sets is an area of ongoing work, but beyond the scope of this proof-of-principle study. Finally, the field of automated brain metastasis detection is rapidly evolving, with the performance benchmarks of leading models improving steadily. Although it is not the intent of this work to compare the performance of the model described here to external models, determining the importance of anatomic contextual information for novel models will be an important direction for future research.

In conclusion, this work provides proof of the principle that providing anatomic contextual information can improve automated segmentation performance for brain metastases. This opens a new avenue for research that can complement efforts to develop novel segmentation algorithms and suggests opportunities for further development of standardized data sets.

## Disclosures

Ethan Truong reports no conflicts of interest. Jordan Houri reports no conflicts of interest. Gretchen Hermann reports no conflicts of interest. Vitali Moiseenko reports no conflicts of interest. Jeffrey Rudie reports consulting fees, medical advisory board membership, and stock or stock options at Cortechs.ai, as well as stock or stock options in Subtle Medical. Aaron B. Simon reports speaker fees from Icotec Medical, Inc. Jona Hattangadi-Gluth reports no conflicts of interest.

## Appendix Supplementary materials

nnUNet Comprehensive Summary Image, application 1

nnUnet_Supplement Image, application 2

## Footnotes

Supplementary material associated with this article can be found in the online version at doi:10.1016/j.adro.2026.102092.
