# Supplementary material for: Effect of Anatomic Contextual Information on the Performance of a Convolutional Neural Network Tasked With Brain Metastasis Detection
Source: Adv Radiat Oncol. 2026 May 25;11(10):102092. doi: 10.1016/j.adro.2026.102092 (PMC13571451; doi:10.1016/j.adro.2026.102092)
Supplement: Supplementary file 1 [file mmc1.docx]

nnU-NET MODELS Comprehensive Summary

MODEL FRAMEWORK

These models were developed using nnU-Net v1 (MIC-DKFZ implementation),
specifically the nnUNetTrainerV2 framework with nnUNetPlansv2.1 and the
3d_fullres configuration.

Training was performed using a single “all” configuration (no
cross-validation folds). The best-performing checkpoint (model_best)
from the all/ directory was selected for release.

Models were exported using:

nnUNet_export_model_to_zip -t -m 3d_fullres -tr nnUNetTrainerV2 -pl
nnUNetPlansv2.1 -f all –disable_strict

Strict mode was disabled to allow export without requiring a
postprocessing.json file.

The resulting zip archive is inference-ready and can be installed on any
system running nnU-Net v1 using:

nnUNet_install_pretrained_model_from_zip <model.zip>

INFERENCE USAGE

After installation, inference can be performed with:

nnUNet_predict -i -o -t -m 3d_fullres -tr nnUNetTrainerV2 -pl
nnUNetPlansv2.1 -chk model_best

The model was trained without ensemble folds and does not rely on
cross-validation ensembling. It uses the single exported checkpoint
(model_best).

Postprocessing is not enforced during export. If desired, postprocessing
can be determined separately using nnUNet_determine_postprocessing.

CRITICALLY IMPORTANT: INPUT REQUIREMENTS

The model requires TWO input channels per case:

Channel 0 (0000): Current post-contrast T1-weighted MRI (T1post) Channel
1 (0001): Prior metastasis mask image registered into the space of the
current MRI

The second channel encodes previously treated metastases spatially
mapped to the current scan.

This is a REQUIRED input channel.

-   If prior treated metastases exist, the prior segmentation must be
    rigidly or deformably registered into the current image space.
-   If no prior metastases exist, a BLANK (all-zero) image with
    identical dimensions, spacing, orientation, header, and affine
    matrix as the current MRI must still be provided.

Inference may fail or behave unpredictably if the second channel is
omitted.

Images must follow standard nnU-Net channel naming conventions:

CASEID_0000.nii.gz (current T1post) CASEID_0001.nii.gz (registered prior
mask or blank image)

DATA PREPARATION PIPELINE

The provided preprocessing notebooks act as structured wrappers around
the standard nnU-Net v1 CLI workflow. They perform the following steps:

1.  Configure required nnU-Net environment variables:

    -   nnUNet_raw_data_base
    -   nnUNet_preprocessed
    -   RESULTS_FOLDER

2.  Construct the nnU-Net task directory structure:

    -   imagesTr/
    -   labelsTr/
    -   imagesTs/

3.  Copy subject-level NIfTI files into the nnU-Net raw data directory.

4.  Rename files to nnU-Net channel format:

    -   *_0000.nii.gz (T1post)
    -   *_0001.nii.gz (Prior metastasis mask)

5.  Generate dataset.json specifying:

    -   Modalities
    -   Label mappings
    -   Training/test splits

6.  Run preprocessing and planning:

    nnUNet_plan_and_preprocess -t –verify_dataset_integrity

7.  Train using:

    nnUNet_train 3d_fullres nnUNetTrainerV2 Task all –npz

Predictions can optionally be remapped back to original patient
identifiers after inference for downstream evaluation or packaging.

REPRODUCIBILITY DETAILS

nnU-Net version: v1 (nnUNetTrainerV2 era) Configuration: 3d_fullres
Plans: nnUNetPlansv2.1 Trainer: nnUNetTrainerV2 Checkpoint: model_best
Training mode: single “all” configuration (no CV folds) Export mode:
–disable_strict (postprocessing not required)

These models are compatible with standard nnU-Net v1 installations and
do not require custom architecture modifications.
