# Supplementary material for: Effect of Anatomic Contextual Information on the Performance of a Convolutional Neural Network Tasked With Brain Metastasis Detection
Source: Adv Radiat Oncol. 2026 May 25;11(10):102092. doi: 10.1016/j.adro.2026.102092 (PMC13571451; doi:10.1016/j.adro.2026.102092)
Supplement: Supplementary file 2 [file mmc2.docx]

Supplemental Table 1: OAR segmentation Performance for BM+OAR model

| Performance Metric | Performance  median (IQR) | | | | |
| --- | --- | --- | --- | --- | --- |
|  | Brainstem | Globe | Lens | Optic Nerves | Optic Chiasm |
| DSC | 0.92  (0.91-0.93) | 0.89  (0.88-0.92) | 0.63  (0.55-0.73) | 0.70  (0.66-0.73) | 0.68  (0.62-0.74) |
| HD95  (mm) | 1.41  (1.00-1.75) | 1.11  (0.94-1.43) | 1.95  (1.27-2.24) | 1.76  (1.41-2.34) | 2.55  (1.81-3.91) |
| Sensitivity | 1.00  (1.00-1.00) | 1.00  (1.00-1.00) | 1.00  (1.00-1.00) | 1.00  (1.00-1.00) | 1.00  (1.00-1.00) |
| Positive Predictive Value | 1.00  (1.00-1.00) | 1.00  (1.00-1.00) | 1.00  (1.00-1.00) | 1.00  (1.00-1.00) | 1.00  (1.00-1.00) |

**Supplemental Figure 1: Representative OAR segmentation from the BM+OAR model.** Each row representents a unique slice from a single patient. OAR structures are designated by color as follows: Brainstem (green), Optic Chiasm (purple), Globes (blue), Optic Nerves (cyan), Lenses (yellow).
